# Syndromes associated with mitochondrial DNA depletion

**DOI:** 10.1186/1824-7288-40-34

**Published:** 2014-04-03

**Authors:** Célia Nogueira, Ligia S Almeida, Claudia Nesti, Ilaria Pezzini, Arnaldo Videira, Laura Vilarinho, Filippo M Santorelli

**Affiliations:** 1 National Institute of Health, Genetics Department, Research and Development Unit, Porto, Portugal; 2 IRCCS Stella Maris, Molecular Medicine for Neuromuscular and Neurodegenerative Diseases, Pisa, Italy; 3 ICBAS-Instituto de Ciências Biomédicas de Abel Salazar, University of Porto, Porto, Portugal; 4 Department of Child Neurology and Neurogenetics, IRCCS Stella Maris, University of Pisa, via dei Giacinti 2, Calambrone, Pisa 56128, Italy; 5 Newborn Screening Metabolism and Genetics Unit, Department of Genetics, National Institute of Health, Rua Alexandre Herculano, 321, Porto 4000-055, Portugal

**Keywords:** Mitochondrial DNA depletion syndrome, Mitochondrial myopathy, Mitochondrial encephalomyopathy, Hepatocerebral syndrome, mtDNA, OxPhos, Alpers-Huttenlocher syndrome

## Abstract

Mitochondrial dysfunction accounts for a large group of inherited metabolic disorders most of which are due to a dysfunctional mitochondrial respiratory chain (MRC) and, consequently, deficient energy production. MRC function depends on the coordinated expression of both nuclear (nDNA) and mitochondrial (mtDNA) genomes. Thus, mitochondrial diseases can be caused by genetic defects in either the mitochondrial or the nuclear genome, or in the cross-talk between the two. This impaired cross-talk gives rise to so-called nuclear-mitochondrial intergenomic communication disorders, which result in loss or instability of the mitochondrial genome and, in turn, impaired maintenance of qualitative and quantitative mtDNA integrity. In children, most MRC disorders are associated with nuclear gene defects rather than alterations in the mtDNA itself.

The mitochondrial DNA depletion syndromes (MDSs) are a clinically heterogeneous group of disorders with an autosomal recessive pattern of transmission that have onset in infancy or early childhood and are characterized by a reduced number of copies of mtDNA in affected tissues and organs. The MDSs can be divided into least four clinical presentations: hepatocerebral, myopathic, encephalomyopathic and neurogastrointestinal. The focus of this review is to offer an overview of these syndromes, listing the clinical phenotypes, together with their relative frequency, mutational spectrum, and possible insights for improving diagnostic strategies.

## Introduction

Mitochondria, present in almost all eukaryotic cells, are dynamic cellular organelles specifically involved in the production of cellular energy via the mitochondrial respiratory chain (MRC) and the oxidative phosphorylation (OxPhos) system. In addition to their most important function, ATP production, mitochondria are involved in the regulation of other cellular pathways such as calcium homeostasis, apoptosis and programmed cell death [1].

Mitochondrial disorders are a group of genetically and phenotypically pleiomorphic disorders with an estimated incidence of between 1:5,000 and 1:10,000 live births [2]; they are often attributable to OxPhos system dysfunction, which leads to a deficiency in ATP production.

The MRC is regulated through the interaction of two physically and functionally separate genomes: the nuclear DNA (nDNA) and the mitochondrial DNA (mtDNA) genomes. Of the estimated > 1000 proteins making up the mitochondrial proteome [3] (see also http://www.broadinstitute.org/pubs/MitoCarta/human.mitocarta.html), about 92 nDNA-encoded ones are the structural subunits forming the five multiprotein complexes embedded in the inner mitochondrial membrane. Human mtDNA encodes 13 subunits of the OxPhos complex, two ribosomal RNA (rRNA) genes and 22 transfer RNA (tRNA) genes, all of which are required for initiating protein translation and synthesis [4]. Therefore, although human mtDNA codes for the basic machinery of protein synthesis, a number of nuclear-encoded factors (including the enzymes for replication, repair and transcription) are also needed to allow protein translation. This dependency lies at the heart of several recently recognized human diseases that are characterized by secondary abnormalities of mtDNA. Cross-talk between the nDNA and mtDNA genomes is crucial for the maintenance of qualitative and quantitative mtDNA integrity and for correct mitochondrial protein production. Multiple deletions, depletion, or a combination of the two in critical tissues, are “hallmarks” of disease conditions arising from disrupted communication between these two genomes. Since a congruous amount of mtDNA is required to produce the key subunits of MRC complexes, mtDNA depletion will result in organ dysfunction due to insufficient synthesis of the respiratory chain components needed for adequate energy production [5].

**Figure 1:**
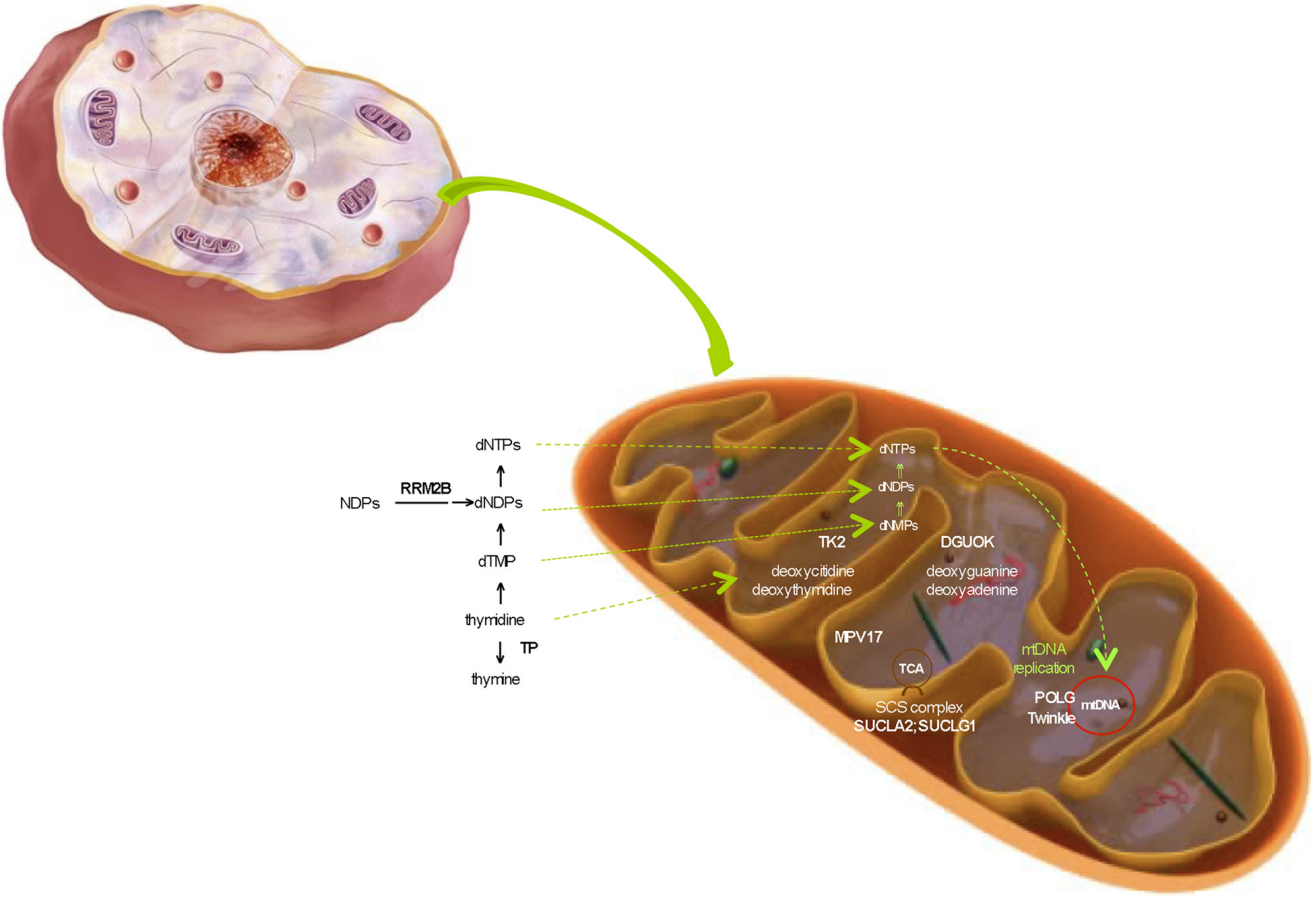
**Schematic view of the mitochondrion and the genes involved in mitochondrial depletion syndromes.** A “magnifying lens” view of the genes (namely, *POLG* and *C10orf2*-Twinkle) thought to be involved in the replication of mitochondrial DNA (mtDNA), those thought to be important in the metabolism of the mitochondrial deoxynucleotide (dNTP) pool (via progressive phosphorylation of deoxythymidine, deoxycytidine, deoxytadenine, and deoxiguanosine), and those involved in the tricarboxylic acid cycle and secondary OxPhos involvement.

The mtDNA depletion syndromes (MDSs) are a heterogeneous group of autosomal recessive disorders, characterized by low mtDNA levels in specific tissues. These syndromes are a consequence of defects in mtDNA maintenance caused by mutations in nuclear genes involved in either nucleotide synthesis (*TK2, SUCLA2, SUCLG1, RRM2B, DGUOK, MPV17* and *TYMP*) or mtDNA replication (*POLG, C10orf2*). The first of the above groups of genes produces proteins that maintain the mitochondrial deoxynucleotide triphosphate (dNTP) pool; dNTPs can be synthesized via either the *de novo* pathway (cell cycle-regulated) or the salvage pathway (in which their production involves the utilization of preexisting deoxynucleosides to synthesize DNA precursors). Given that mtDNA replicates continuously and independently of cell division, mutations in any of the genes responsible for maintaining the dNTP pool will result in mtDNA depletion (Figure 1). Mutations in *POLG*, the gene encoding the DNA polymerase gamma (POL γ), which is required for replication and repair of mtDNA, as well as mutations in *C10orf2* (Twinkle), a mitochondrial helicase, result in impaired mitochondrial protein synthesis and an incapacity to supply sufficient mtDNA to daughter cells during cell divisions; this, in turn, leads to a reduction of mitochondrial genome content [6].

The MDSs are rare, devastating diseases that manifest typically, although not exclusively, soon after birth, and usually lead to death in infancy or early childhood. Although they often manifest themselves in a tissue-specific manner [7], it is not unknown for multiple organs, including the heart, brain, and kidneys, to be affected [8].

This paper sets out to provide an overview of the clinical manifestations and molecular etiologies of the nuclear defects involved in MDSs and to offer possible insights for improving diagnostic strategies.

### The mtDNA depletion syndromes – clinical manifestations

The MDSs are usually classified as myopathic, encephalomyopathic, hepatocerebral or neurogastrointestinal [9]. Table 1 summarizes the main clinical manifestations and molecular etiologies associated with these different forms.

**Table 1:** **Relative frequency of mutations associated with the different mitochondrial DNA depletion syndromes (MDS, source: HGMD Professional database: **http://www.hgmd.cf.ac.uk/**)**

**Mitochondrial DNA depletion syndromes**	**Period of onset**	**Clinical features**	**Genes (aliases)**	**Chromosomal *LOCI***	**Numbers of mutations**	References*
Hepatocerebral mtDNA depletion syndrome	Neonatal, early childhood	Hepatic dysfunction; psychomotor delay; hypotonia; lactic acidosis; nystagmus; neurological dysfunction	*POLG (Polg1/PolgA)*	15q25	8	[10]
			*C10orf2 (Twinkle/PEO1)*	10q24	3	[11]
			*DGUOK (dGK)*	2p13	51	[12]
			*MPV17 (SYM1)*	2p23.3	28	[13]
			*TK2*	16q22-q23.1	1	[14]
Alpers-Huttenlocher syndrome	Early childhood	Hepatic dysfunction; *epilepsia partialis continua*; neurological dysfunction	*POLG (Polg1/PolgA)*	15q25	54	[15]
Myopathic mtDNA depletion syndrome	Infancy, early childhood	Hypotonia; muscle weakness; dysarthria and dysphagia; failure to thrive	*POLG (Polg1/PolgA)*	15q25	1	[5]
			*TK2*	16q22-q23.1	34	[16]
			*RRM2B (p53R2)*	8q23.1	3	[5]
			*DGUOK (dGK)*	2p13	1	[17]
Encephalomyopathic mtDNA depletion syndrome	Infancy	Hypotonia; muscle weakness; psychomotor delay; sensorineural hearing impairment; lactic acidosis; neurological dysfunction	*RRM2B (p53R2)*	8q23.1	14	[18]
			*TK2*	16q22-q23.1	1	[19]
			*SUCLA2*	13q12.2	9	[20]
			*SUCLG1*	2p11.3	13	[21]
Mitochondrial neurogastrointestinal encephalomyopathy	Late childhood, adolescence	Gastrointestinal dysmotility; weight loss; peripheral neuropathy;ptosis; neurological dysfunction	*TYMP (ECGF1)*	22q13	81	[22]
			*RRM2B (p53R2)*	8q23.1	2	[23]
			*POLG (Polg1/PolgA)*	15q25	1	[24]

MDS are inherited in an autosomal recessive pattern; mutations in *POLG* gene outside MDS may be associated with autosomal recessive/dominant pattern of inheritance. *References of the first mutations published associated with the phenotype.

#### Hepatocerebral MDS

Hepatocerebral MDS encompasses relatively common clinical conditions which, to date, have been associated with over 50 different mutations, including variants in the *POLG*, *C10orf2*, *TK2*, *DGUOK*, *MPV17* and *SUCLG1* genes. Their onset occurs within the first six months of life and affected subjects usually die within one year of onset. Common symptoms include persistent vomiting, failure to thrive, hypotonia and hypoglycemia associated with progressive neurological deterioration. Histological changes on liver biopsy include fatty degeneration, bile duct proliferation, fibrosis and collapse of lobular architecture. Reduced cytochome c oxidase (COX) histochemistry and combined deficiency of mtDNA-encoded MRC complexes have been detected in the livers of infants and toddlers. A peculiar form of liver failure occurs in Alpers-Huttenlocher syndrome (AHS), an early-onset, fatal disease that is, in addition, characterized by intractable seizures that evolve into *epilepsia partialis continua*, and by global neurological deterioration. The liver dysfunction is usually progressive as well, evolving from microvesicular steatosis with bile duct proliferation into cirrhosis and organ failure [25,26]. Brain MRI includes signal abnormalities in the basal ganglia and thalami, irregularly widened ventricles and partial pachygyria. Patients usually present MRC deficiencies and low mtDNA in the liver, although both these markers may be normal in skeletal muscle. The prognosis is severe [27].

#### i) DGUOK

The deoxyguanosine kinase (*DGUOK*) gene lies on chromosome 2p13, and codes for a 2-deoxyribonucleoside enzyme that catalyzes the first step in the mitochondrial deoxypurine salvage pathway. More specifically, the DGUOK enzyme catalyzes the phosphorylation of purine deoxyribonucleosides into the corresponding nucleotides (deoxyguanosine and deoxyadenosine) necessary for the maintenance of mitochondrial dNTP pools [8,28]. The typical phenotype associated with mutations in *DGUOK* is neonatal onset of progressive liver disease and feeding difficulties, usually associated with hypotonia, nystagmus, and psychomotor retardation by the age of three months. Most cases harbor null mutations and die before the age of two years. Peripheral neuropathy and renal tubulopathy have occasionally been reported [29]. Depletion of mtDNA has been documented mainly in the liver, where it results in combined reduction of complexes I, III and IV; the amount of mtDNA is usually normal in other tissues, at least at disease onset. Histological analyses of liver biopsies show variable findings, including microvesicular steatosis and cholestasis. Progression is usually rapid and life expectancy is low [30]. The majority of affected infants show an elevated serum concentration of tyrosine or phenylalanine on newborn screening. Intrahepatic cholestasis typically includes elevation of liver transaminases, gamma-glutamyltransferase and conjugated hyperbilirubinemia. An increased serum concentration of ferritin is often observed [31,32].

Since the initial report of pathogenic mutations in 2001 [12], more than 100 patients have been reported harboring over 50 different *DGUOK* mutations (fully reviewed in [9]).

#### ii) MPV17

The *MPV17* gene is located on chromosome 2p23.3 and encodes a mitochondrial inner membrane protein whose function is not yet completely characterized, even though its key role in controlling mtDNA maintenance and OxPhos activities in mammals and yeast is well established [33]. The clinical presentation associated with mutations in *MPV17* consists of severe liver failure, hypoglycemia, growth retardation, neurological symptoms and multiple brain lesions during the first year of life [34]. Marked liver depletion is found in association with biochemical deficits, with complex I or complexes I + III being most affected. Both mildly reduced mtDNA content and impaired OxPhos activities may also be noted in muscle [35]. Histological analyses of the liver have revealed swollen granular hepatocytes, and steatosis with focal pericellular and periportal fibrosis. More than 30 patients with mutations in *MPV17* have been reported [9], while over 20 different mutations have been described in infantile-onset hepatocerebral syndrome and also in Navajo neurohepatopathy, an autosomal recessive multisystem disorder prevalent in the Navajo community in the Southwestern United States [28]. There exist three main subtypes: infantile (onset < 6 months) and childhood (< 5 years) forms, characterized by hypoglycemic episodes and severe progressive liver dysfunction requiring liver transplantation, and a “classic” form characterized by moderate hepatopathy and progressive motor and sensory axonal neuropathy. The three subtypes are also associated with variable degrees of demyelination in both the central and the peripheral nervous systems.

#### iii) POLG

The *POLG* gene lies on chromosome 15q24 and encodes POL γ, the only DNA polymerase responsible for mtDNA replication and repair in mitochondria. POL γ is composed of a catalytic subunit that has both polymerase and proofreading exonuclease activities, and an accessory subunit, which increases enzyme processivity [32]. This holoenzyme functions in conjunction with the mtDNA helicase and the mitochondrial single-stranded DNA-binding protein to form the minimal replication apparatus [36].

Over 200 mutations have been reported in the *POLG* gene (Human DNA Polymerase Gamma Mutation database [37]), making this gene a hot-spot for mutations associated with mitochondrial diseases [38,39]. Individuals affected by POLG-related disorders present with a large variety of clinical phenotypes, ranging from autosomal dominantly and recessively inherited forms of progressive external ophthalmoplegia (PEO) to juvenile spinocerebellar ataxia and epilepsy with or without dysarthria, and AHS [27,40].

Approximately 45 different point mutations in *POLG* cause AHS [27] whose incidence has been estimated to be ~1:50,000 [41]. The two most common *POLG* mutations detected in AHS, i.e., p.Ala467Thr and p.Trp748Ser, can be either homozygous or heterozygous and can be present in combination with other variants. Carrier frequency for these mutations is higher in Western countries than elsewhere. For example, in Finland it is 1:125 for p.Trp748Ser, in Norway it is 1:50, if both variants are combined, whereas 0.6% of the control Belgian population harbors p.Ala467Thr. A single ancestral founder mutation is hypothesized for both variants [6].

#### iv) *C10orf2* (*Twinkle*)

The mitochondrial protein Twinkle, encoded by *C10orf2/PEO1* located on chromosome 10q24, is an mtDNA helicase, active as a homohexamer and bound to mtDNA in mitochondrial nucleoids [42]. Mutations in *C10orf2* cause dominantly inherited disorders, such as pure adult-onset PEO with multiple mtDNA deletions, or recessive clinical conditions including severe early-onset hepato-encephalopathy or infantile-onset spinocerebellar ataxia (IOSCA) and low mtDNA in the brain and liver, but not in skeletal muscle [6]. Neuroimaging can show cortical cerebellar atrophy; OxPhos assays show reduction of complexes I, III and IV.

Infantile-onset spinocerebellar ataxia is a severe autosomal recessive neurodegenerative disorder that manifests itself after 9-18 months of age through progressive atrophy of the cerebellum, brain stem and spinal cord, ataxia during the first two years of life, hypotonia with sensory axonal neuropathy, optic atrophy, hearing impairment and ophthalmoplegia [6]. Patients usually survive to adulthood. The severe neurological phenotype and the observed absence of muscle involvement in IOSCA suggest that Twinkle may play a crucial role in the maintenance and function of specific neuronal subpopulations [8].

Infantile-onset spinocerebellar ataxia is the second most common heritable ataxia in Finland, this high frequency being due to the founder effect of the p.Tyr508Cys variant and a carrier frequency of about 1:200. The same mutation has also been described in cases with severe epileptic encephalopathy and hepatic failure [43].

#### Myopathic MDS

The symptoms of myopathic MDS usually appear in the first year of life and consist of feeding difficulties, failure to thrive, hypotonia, muscle weakness and, occasionally, PEO. Death is often due to pulmonary insufficiency and recurrent infections, but some patients survive into their teens [44]. Muscle biopsy may show proliferation of mitochondria, and patchy or diffuse deficiency of COX. Biochemical defects are always present in all mtDNA-related respiratory chain complexes in muscle mitochondria. Serum creatine kinase levels may be variably elevated [45].

#### i) TK2

The *TK2* gene lies on chromosome 16q22 and encodes thymidine kinase (TK2). TK2 is an intramitochondrial pyrimidine nucleoside kinase that phosphorylates dNTPs, such as deoxythymidine, deoxycytidine and deoxyuridine, thereby participating in the salvage pathway of deoxynucleotide synthesis [46]. Mitochondrial dNTP pools arise either through active transport of cytosolic dNTPs or through salvage pathways. Both pathways are essential for the replication of mtDNA, since the mitochondrion is unable to synthesize dNTPs *de novo.* Mutations in *TK2* primarily affect muscle tissue, and have little or no effect on the liver, brain, heart or skin. The clinical presentation of TK2-related MDS is variable, with a broad phenotype. Typical manifestations include a severe, rapidly progressing myopathy of infantile or childhood onset. The disease course is rapidly progressive, leading to respiratory failure and death within months or a few years, although milder phenotypes with slower progression and longer survival have been described [6]. To date, around 50 individuals with *TK2*-related MDS have been reported [9]. Since the description of the first mutation in 2001 [16], 31 different pathogenic autosomal recessive mutations have been described, and the different phenotypes may be explained by variable degrees of residual activity of the mutant enzymes. Mutations in *POLG* and *RRM2B* are additional etiologies in myopathic presentations of reduced mitochondrial copy number. Milder presentations manifest as late-onset proximal muscle weakness or adult-onset progressive myopathy, with or without sensorineural hearing loss [9].

#### Encephalomyopathic MDS

Encephalomyopathic MDS embraces phenotypes characterized by infantile onset of hypotonia with severe psychomotor retardation, high blood lactate levels, progressive hyperkinetic-dystonic disorder, external ophthalmoplegia, deafness, generalized seizures and variable renal tubular dysfunction. Brain MRI is often abnormal and was initially suggested to be reminiscent of the pathological features seen in Leigh syndrome [8].

#### i) RRM2B

The *RRM2B* gene lies on chromosome 8q23.1 and codes for the small subunit of p53-inducible ribonucleotide reductase, a heterotetrameric enzyme responsible for *de novo* conversion of ribonucleoside diphosphates into the corresponding deoxyribonucleoside diphosphates that are crucial for DNA synthesis [18]. Ribonucleotide reductase is the main regulator of the nucleotide pools in the cytoplasm and its small subunit is expressed in post-mitotic cells, where it probably has a key function in maintaining the mitochondrial dNTP pools for mtDNA synthesis. Mutations in *RRM2B* usually result in neonatal hypotonia, lactic acidosis, failure to thrive and tubulopathy. Psychomotor delay, sensorineural hearing loss and a profound reduction of mtDNA copy numbers in skeletal muscle [18] are also present. The disease has a rapid progression and leads to death within a few months of onset. The complex associated phenotype suggests that the consequences of defective mitochondrial dNTP pools can vary dramatically depending on the residual amount of functional enzyme. Approximately 15 affected infants have been described [9]. Of the 31 mutations described to date, 30 are associated with major phenotypes.

#### ii) *SUCLA2* and *SUCLG1*

Succinyl CoA synthase is a mitochondrial matrix enzyme that catalyzes the reversible synthesis of succinate and ATP or GTP from succinyl-CoA and ADP in the tricarboxylic acid cycle. It is made up of two subunits, *alpha* and *beta*, encoded by *SUCLG1* on chromosome 2p11.3 and *SUCLA2* on 13q12.2-q13.3, respectively. Mutations in *SUCLA2* (coding for succinate-CoA ligase, *beta* subunit) and *SUCLG1* (coding for succinate-CoA ligase, *alpha* subunit) cause an encephalomyopathic form of infantile MDS, but mutations in *SUCLG1* can also cause a severe disorder characterized by antenatal dysmorphisms, neonatal metabolic crisis, and early death [47]. Differences in presentation between patients might depend on differences in the residual amounts of the protein [20,48,49]. Useful diagnostic clues in succinyl CoA synthase disorders are the presence of “mildly” elevated urinary methylmalonic acid, found in all patients, and the presence of tricarboxylic acid cycle intermediates (methylcitrate, lactate, carnitine esters, 3-hydroxyisovalericacid), found in most cases. Some patients die as infants, but others survive longer. The clinical features include early childhood hypotonia, developmental delay and, almost invariably, progressive dystonia and sensorineural deafness. Mutations in *SUCLA2* and *SUCLG1* seem to disrupt the association between succinyl CoA synthase and mitochondrial nucleoside diphosphate kinase, resulting in mitochondrial dNTP pool imbalance and, eventually, low levels of mtDNA in muscle [21]. Twenty-three mutations were recently reported in a series of 54 individuals [9]: 10 in the 34 subjects with mutated *SUCLA2* and 13 in the 20 with mutated *SUCLG1*.

#### Neurogastrointestinal MDS

Mitochondrial neurogastrointestinal encephalomyopathy (MNGIE) is an autosomal recessive disorder clinically characterized by onset between the first and fifth decades of life, although in the vast majority of cases onset occurs before the age of 20 years. All affected individuals develop weight loss and progressive gastrointestinal dysmotility manifesting as early satiety, nausea, dysphagia, gastroesophageal reflux, postprandial emesis, episodic abdominal pain with distention, and diarrhea. In addition, all affected individuals have motor and sensory demyelinating neuropathy, in some cases accompanied by axonal neuropathy. The neuropathy typically presents with distal weakness and paresthesias, showing a symmetric stocking-glove distribution. Ptosis and ophthalmoplegia are common.

Affected individuals can have elevated CSF protein and plasma lactate. Thymidine and deoxyuridine are increased in plasma. Thymidine phosphorylase (TP) enzyme activity in leukocytes is usually less than 10% of the control mean. Neuroimaging typically demonstrates diffuse white matter changes [9].

In MNGIE, mtDNA abnormalities can include depletion, multiple deletions and point mutations [50]. Mutations in *TYMP* and *RRM2B* have been linked to MNGIE, although variants in *POLG* have recently been detected in conditions mimicking MNGIE (so-called MNGIE-like syndromes) [24].

Skeletal muscle generally shows ragged-red fibers and defects in single or multiple OxPhos complexes, especially COX. However, MNGIE has also been reported without morphological, enzymatic, or mtDNA changes in skeletal muscle. Life expectancy is reduced (ranging from 25–60 years) [9].

#### i) *TYMP*

The *TYMP* gene is located on chromosome 22q13 and encodes the cytosolic TP enzyme, which catalyzes the conversion of thymidine to thymine and of deoxyuridine to uracil and is therefore essential for pyrimidine catabolism. TP deficiency causes systemic accumulation of thymidine and deoxyuridine; this leads to deoxynucleotide pool imbalance and mtDNA instability, in turn resulting in the presence of multiple deletions and partial depletion of muscle mtDNA [22].

The first pathogenic mutations in the *TYMP* gene were described in 1999 [22] and since then over 70 mutations have been described, most associated with MNGIE.

### MDS - diagnostic approaches

**Figure 2:**
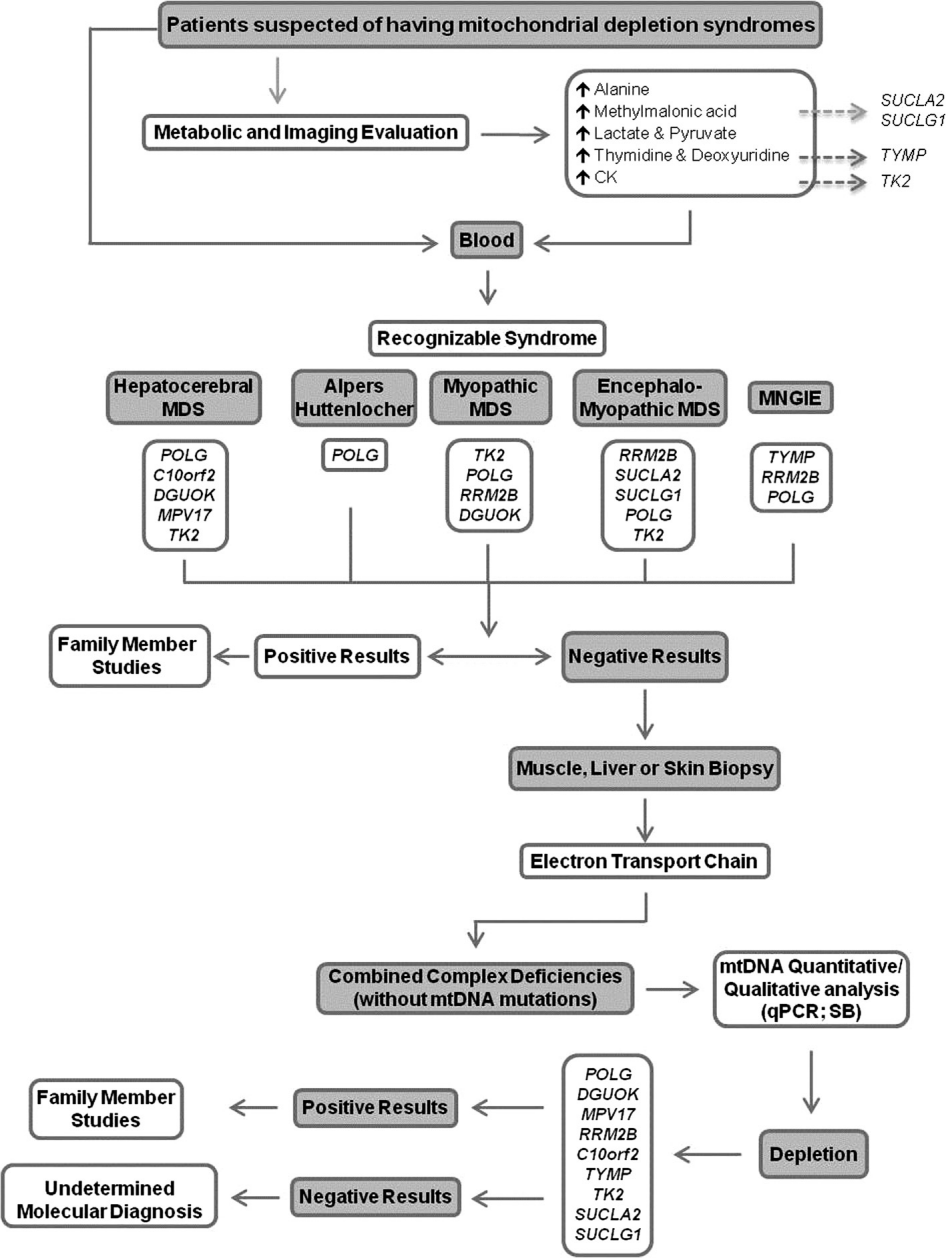
**Diagnostic algorithm for mitochondrial depletion syndromes, based on clinical and biochemical information.** Abbreviations: *MDS***-** mitochondrial depletion syndrome; *CK***-** creatine kinase; *MNGIE***-** mitochondrial neurogastroIntestinal encephalomyopathy; *qPCR***-** real-time PCR; *SB***-** Southern blot.

The suspicion of MDS is usually based on the clinical presentation, which may range from well-defined syndromes to non-specific multisystem phenotypes, and usually includes neurological involvement. Establishing a specific MDS diagnosis is challenging and requires the integration of clinical assessments, family history, biochemical testing and histopathological examination in affected tissues. It is important to obtain the appropriate biochemical and clinical information before starting any molecular investigations in order to increase the chances of a successful molecular diagnosis. Biochemical determination of MRC complexes is also important, although the results can be normal if skeletal muscle is not among the affected tissues. Quantitative real-time PCR quantification of total mtDNA content in affected tissues, using a nuclear gene as reference, is a prerequisite for correct interpretation of the amount of mtDNA present, although it is important, given the dynamic nature of mtDNA copies in different ages and tissues, to select appropriate age-matched control materials [51]. A reduction in mtDNA copy number to 60-65% of the average recorded in age-matched controls is the empirical cut-off level for a diagnosis of primary MDS. However, the reduction could be even greater, with mtDNA levels in most patients being about 20-25% of age-matched normal controls. Biochemical data, such as lactate, pyruvate, alanine and organic acid profiles, as well as neuroimaging findings, are also important diagnostic clues. Serum CK is elevated particularly when mutations occur in *TK2*; serum thymidine is impaired in *TYMP*, and mildly elevated levels of urinary methylmalonic acid and methylcitrate occur in disorders linked to *SUCLA2* or *SUCLG1*[6]. Figure 2 summarizes the diagnostic algorithm in syndromes associated with mitochondrial DNA depletion. Reaching a full molecular characterization is also important for adopting appropriate therapies: the detection of changes in *POLG* and *C10orf2* in toddlers with severe drug-resistant epilepsy should prompt consideration of the risk of valproate (VPA)-induced liver toxicity [52].

### MDS - therapeutic considerations

The management of mitochondrial diseases is largely supportive, given that there exists no “magic pill” [53]. Palliative treatments with vitamins, cofactors and respiratory substrates have been used, but they showed poor efficacy. In recent years several approaches have been adopted, mostly involving modulation of the different pathways regulating mitochondrial biogenesis [39], but they have yet to be applied in the clinic. Meanwhile, some therapeutic avenues have been tried in clinical practice, although none has shown evidence-based efficacy.

Liver transplantation may be beneficial to patients with hepatopathy caused by *DGUOK* mutations if no neurological symptoms have developed. However, it would be contraindicated in the presence of significant hypotonia, psychomotor retardation or nystagmus [31]. In patients with *MPV17*, and in VPA-induced organ failure, transplantation has increased quality of life and life expectancy in some patients [5,54], but liver-transplanted children may go on to develop neurological symptoms [55,56]. A controlled diet, avoiding hypoglycemia, has been proposed to slow down disease progression and allow supportive care [57]. Some improvement was suggested with succinate or coenzyme Q10 together with a lipid-rich diet [58]. Furthermore, folate levels may be deficient in the CSF of some patients and detection of low folate in the CSF may prompt replacement therapy [59]. Levocarnitine, creatine monohydrate, coenzyme Q10, B vitamins, and antioxidants, such as alpha lipoic acid, vitamin E, and vitamin C, have often been used as pro-energy supplements in mitochondrial disorders in general and in MDS in particular, but longer follow-ups are necessary to evaluate the opportuneness of recommending such dietary interventions [53]. In MNGIE, a correlation between plasma thymidine levels and the severity of the phenotype has been observed [60]. Therefore, attempts to reduce the circulating nucleotide levels could result in disease improvement. Enzyme replacement therapy has been used in MNGIE: infusions of platelets from healthy donors reduced circulating thymidine and deoxyuracile levels and partially restored TP activity. The limitation of this therapy was the short half-life of the platelets [61]. Allogeneic stem cell transfusions have been given to two patients with MNGIE [62,63]; although more experience is needed to illustrate the clinical benefit of this treatment, it opens up a therapeutic possibility for disorders of nucleoside metabolism. Finally, continuous ambulatory peritoneal dialysis has also been used in MNGIE to reduce thymidine levels, and this treatment improved symptoms during a three-year follow-up [64].

## Concluding remarks

A mitochondrial disease manifesting at, or soon after, birth is more likely to be associated with nDNA than with mtDNA mutations [39], but until very recently our ignorance regarding the mechanisms underlying mitochondrial gene transcription and translation and the complex interaction between the “two genomes” limited our diagnostic power.

Mitochondrial DNA depletion, which can result from any imbalance in the mitochondrial nucleotide pool available for mtDNA replication, as well as abnormalities in mitochondrial replication machinery, has become an increasingly important cause of a wide spectrum of infantile and childhood-onset tissue-specific and multisystem disorders [65]. Consistent with the different phenotypes, mtDNA depletion may affect a specific tissue type (most commonly brain and muscle or liver tissue) or multiple organs, including the heart and kidneys. More than 75% of MDS patients develop full-blown disease within the first year of life, and it is rapidly fatal in most cases [11,66]. Identifying the causative genes is important not only to allow adequate antenatal options, family planning and prenatal diagnosis, but also to improve understanding of the disease pathophysiology and, therefore, improve therapeutic options. From this perspective, the recent advances in the clinical use of next generation sequencing (NGS) technologies will likely facilitate molecular diagnosis of these conditions in the coming years [67-69]. Since NGS is becoming a feasible option in several Mendelian disorders and inborn errors of metabolism [70], it promises to allow the identification of a greater number of patients with mitochondrial disorders as well [71,72]. This would likely resolve some of the open issues emerging from clinical practice in this field, which include difficulties in diagnosing the conditions and in providing adequate counseling, and unpredictable prognoses. An accurate and focused diagnostic workup would also save health-related resources and family distress. Only by achieving a full understanding of the molecular basis of MDS will we be able to gather insights for novel and effective therapeutic strategies.

## Competing interests

The authors declare no conflicts of interest for the present paper.

## Authors’ contributions

All the authors have made substantial contributions to conception and design of the review. All the authors have been involved in drafting the manuscript and revising it critically. All the authors read and approved the final manuscript.
